# Using diffusion distances for flexible molecular shape comparison

**DOI:** 10.1186/1471-2105-11-480

**Published:** 2010-09-24

**Authors:** Yu-Shen Liu, Qi Li, Guo-Qin Zheng, Karthik Ramani, William Benjamin

**Affiliations:** 1 School of Software, Tsinghua University, Beijing 100084, China; 2 Key Laboratory for Information System Security, Ministry of Education of China, Beijing, China; 3 Tsinghua National Laboratory for Information Science and Technology, Beijing, China; 4 Purdue University, West Lafayette, IN, USA

## Abstract

**Background:**

Many molecules are flexible and undergo significant shape deformation as part of their function, and yet most existing molecular shape comparison (MSC) methods treat them as rigid bodies, which may lead to incorrect shape recognition.

**Results:**

In this paper, we present a new shape descriptor, named Diffusion Distance Shape Descriptor (DDSD), for comparing 3D shapes of flexible molecules. The diffusion distance in our work is considered as an average length of paths connecting two landmark points on the molecular shape in a sense of inner distances. The diffusion distance is robust to flexible shape deformation, in particular to topological changes, and it reflects well the molecular structure and deformation without explicit decomposition. Our DDSD is stored as a histogram which is a probability distribution of diffusion distances between all sample point pairs on the molecular surface. Finally, the problem of flexible MSC is reduced to comparison of DDSD histograms.

**Conclusions:**

We illustrate that DDSD is insensitive to shape deformation of flexible molecules and more effective at capturing molecular structures than traditional shape descriptors. The presented algorithm is robust and does not require any prior knowledge of the flexible regions.

## Background

The geometrical shape of a molecule is a key factor for biological activity in computer aided molecular design, rational drug design, molecular docking and function prediction [1]. To exploit the shape similarity between molecules, a useful tool is molecular shape comparison (MSC) that compares the shapes of two or more molecules and identifies common spatial features [1-4]. In computer aided drug design, for instance, an alternative process of *virtual screening *takes advantage of such comparison for searching a molecular database for compounds that most closely resemble a given query molecule [2,3,5,6]. The underlying assumption is that the molecules similar to the active query molecule are likely to share similar properties. One main advantage of MSC is that the molecules with shape similarity can be found without any specification of chemical structure. However, the efficient MSC is still a challenge [1-4] due to the high complexity of 3D molecular shapes.

Although many researchers have proposed various methods for shape comparison of molecules [2,3,7-11], most of them only concerned comparison of 3D rigid objects, little attention has been paid on the deformed shapes of flexible objects. Nevertheless, many molecules are flexible, and this flexibility is often part of their function, which may lead to significant shape changes. Shape similarities between molecules can be missed when different conformations of the same molecule are compared to each other as rigid bodies. Several recent methods [12,13] were proposed for addressing this problem by regarding molecules as flexible shapes, but these methods can not handle well shape deformation of molecules with topological changes. In this paper we developed a new technique for comparing molecular shapes, which is insensitive to shape deformation of flexible molecules, in particular to topological changes.

### Methods of molecular shape comparison

The MSC methods can be roughly divided into two categories [2,3]. One category of MSC, called *superposition *methods, relies on finding the optimal superposition/alignment of two or more molecules compared [1,4,14-16]. The superposition methods usually compare molecular shapes in a particular coordinate system by a priori superposition/alignment, which is non-trivial to achieve robustly. Another category of MSC, called *descriptor *methods, are independent of molecular orientation and position by using descriptor to represent the shape of molecule. The descriptor methods compute the similarity score by comparing the corresponding descriptors between two molecular shapes without any superposition. A 3D shape descriptor, or named *signature*, is a compact representation for some essence of the shape. The shape descriptor is often used as an index in a database of shapes and it enables fast queries and retrieval.

The descriptor methods are simpler and much faster than the traditional superposition methods. Several recent works related to MSC using shape descriptors have been developed such as shape distribution, spherical harmonic descriptor, and 3D Zernike descriptor [5,7,9,17-22]. These descriptors are rigid-body-transformation invariant, and they are often effective for capturing rigid objects. Nevertheless, most of these methods are not deformation invariant and they can not support flexible molecular shape comparison. Deformation invariant representation of nonrigid or flexible shape is a challenging problem in the field of shape analysis. Several recent works focus on the problem of comparing non-rigid shapes [23-27] in computer vision, computer graphics, and pattern recognition. However, these existing descriptors do not perform well for flexible molecules due to their complex shape deformation. It is beyond the scope of the presented paper to give a detailed review of all the existing work; we will only review those most relevant results. The reader may consult [2,3,13] for a general introduction to the MSC problem.

### Methods based on distance descriptors

The distance descriptor between sampling point pairs on shape surfaces may be the simplest and most widely used shape descriptor in 3D shape retrieval. The presented paper also belongs to this category. We first introduce three representative distance descriptors: *Euclidean distance *(ED), *geodesic distance *(GD) and *inner distance *(ID).

#### Euclidean distance

The ED descriptor [20] (also called D2) is usually represented by a histogram of distance values. It consists of three steps for computing the ED descriptor. First, some point pairs are randomly selected on the shape surface, and the Euclidean distances between all sampled point pairs are computed, finally the histogram of all distance distribution is built. The similarity scores between shapes are measured as the differences between their corresponding histograms. The ED descriptor has several advantages. It is both rotation and translation invariant, is computationally cheap (both for generating the descriptor and comparing two descriptors), and describes the overall shape, which means that it is not easily affected by minor shape distortions. Nevertheless, the ED histogram can not capture the shape similarity of flexible objects.

#### Geodesic distance

To overcome the drawback of ED, one can simply apply the GD [23] as the shape descriptor instead of the ED. The GD between two points on a surface is measured as the length of the shortest path along the boundary surface. Although the GD is invariant to surface bending, it is not enable to capture shape articulation deformation well [28], that exists commonly in macromolecular movements (such as the popular hinge motion).

#### Inner distance

In order to overcome the disadvantages of ED and GD, we recently proposed a new shape descriptor based on inner distance (ID) for comparing the shapes of flexible molecules [13]. The ID is the length of the shortest path between landmark points within the molecular shape and it reflects the molecular structure and deformation much better than other distance measures. However, the above distances only measure the shortest path between two points in a sense of the single path, and they may be significantly affected by topological changes of shape deformation.

**Figure 1:**
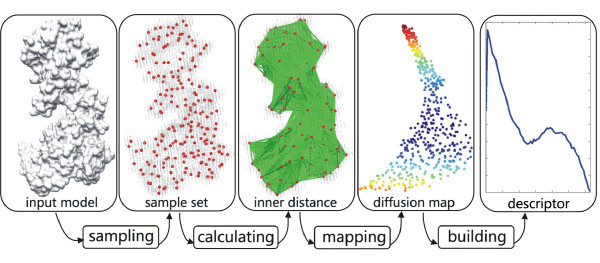
**Flowchart of our method**. Given a molecular shape, four independent steps contain sampling (red points), calculating inner distances (green line segments) between all sample point pairs, computing diffusion distances based on diffusion maps, and building the descriptor (blue histogram). Here the input shape is the volumetric data with the simulated 8 Å resolution density map for GroEL (PDB code: 1AON).

In order to deal with shape deformation of flexible molecules, possibly with topological changes, we propose to use the *diffusion distance *(DD) as a new shape descriptor. The diffusion distance is related to the probability of travelling on the surface from one point to another in a fixed number of random steps [29-31]. The diffusion distance is an average length of paths connecting two points on the shape, while the GD or ID is the length of a shortest path. This naturally makes the diffusion distance less sensitive to topological changes. In our implementation, we combine diffusion distances with the help of inner distances for constructing a new shape descriptor, called Diffusion Distance Shape Descriptor (DDSD), which leads to an average length of paths in a sense of inner distances. The new descriptor is insensitive to shape deformation of flexible molecules, in particular to topological changes, and it is more effective at capturing molecular structures than traditional shape descriptors. Our DDSD is stored as a histogram which is a probability distribution of diffusion distances. Our approach reduces the 3D shape comparison problem of flexible molecules to comparison of DDSD histograms. The core procedure can be divided into four steps: sampling, calculating inner distances, computing diffusion distances based on diffusion maps, and building descriptors (see Figure 1).

**Figure 2:**
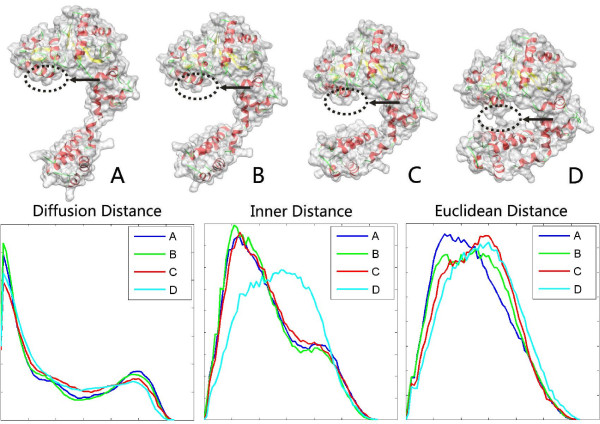
**Our diffusion distance (DD) descriptor is compared to inner distance (ID) and Euclidean distance (ED)**. The first row shows the input four molecules with the same main chain orientation but with different surface shapes, where the arrows mark topological changes. The second row shows the DD, ID and ED descriptors. In each plot, the horizontal x-axis denotes pairwise distances, the vertical y-axis represents distance distributions, and the scale is normalized for the comparison process. Note that DD is not sensitive to shape deformation, in particular to topological changes, so four histograms are closed; in contrast, ED is strongly sensitive to deformation and ID is sensitive to topological changes.

Figure 2 illustrates comparison among three methods: DD, ID, and rigid methods (ED). The first row shows the input four molecules (A, B, C and D) with the same main chain orientation but with different surface shapes. Two source molecules are two conformations of rat DNA polymerase beta: 1BPD (molecule A) and 2BPG (molecule D). Two middle molecules (B and C) are the morph deformation between the two conformations. The deformation between four molecules can be explained by a movement that fixes the top domain of molecule A and bends its bottom domain. Imagine the regions highlighted by arrows. If the surfaces of two domains are not touching, then both inner distance and diffusion distance between two points on the two regions travel throughout the whole molecular shapes. Yet, if the bottom domain is bent so that the surfaces on two domains touch each other, as highlighted in molecule D, the minimal inner distance will "re-route" itself through the "shortcut" across the highlight region instead of going though the whole molecular shapes, leading to a significant change in the inner distance. For the diffusion distance, the new path added as a result of the topological change is averaged with the other paths, which reduces the effect of such a change. The second row in Figure 2 shows comparison among three descriptors: DD, ID and ED. The ED is strongly sensitive to shape deformation, so it is not suitable for flexible molecular shape comparison. Although the ID descriptors can keep almost consistent histogram for the first three deformed molecules, but it is sensitive to shape topological changes, leading to a significant change for molecule D. Note that our DD descriptors reduce the effect of topological changes and remains largely consistent for the four deformed conformation shapes of the same protein.

## Methods

In this section, we first review diffusion distances and inner distances. By combining them, we then present a new method for computing the DDSD of flexible molecules.

### Review of diffusion distances

We recall diffusion distances as described in [29-35]. Coifman et al. introduced diffusion maps and diffusion distances as a method for data parametrization and dimensionality reduction. A 3D shape can be embedded into the Euclidean space via diffusion maps. The diffusion distance is equal to the Euclidean distance in the embedding space corresponding to diffusion maps. Informally, the diffusion distance is considered as an average length of all the paths connecting two points on the shape, and it is related to the probability of arriving from one point to another in a random walk with a fixed number of steps. This makes the diffusion distance a bending invariant function of the path length and the shape width between two points. Since this distance does not rely on just the single path between two points, it is robust to topological changes.

For the exposition simplicity, we regard the 3D shape as a finite set of 3D points. In the diffusion framework, a nonnegative symmetric *diffusion kernel *function *k*(*x*, *y*), which reflects the similarity between two points *x *and *y*, is first constructed over all pairs of points on the shape. The kernel function can lead to a *n *× *n *matrix *K*, where *n *is the number of available points. The matrix is symmetric and has non-zero values. Next, we define

(1) $$p(x,y)=\frac{k(x,y)}{v(x)},$$

Where *v*(*x*) = ∑_*y *_*k*(*x*, *y*) is the sum of the elements in each row. Since we have that *p*(*x*, *y*) ≥ 0 and ∑_*y *_*p*(*x*, *y*) = 1, *p*(*x*, *y*) can be interpreted as the probability for a random walker (Markov process) on the shape to jump from *x *to *y *in a single time step. The corresponding matrix *P *= {*p*(*x*, *y*)} is the transition matrix of this *Markov chain *in a single time step. Note that *p*(*x*, *y*) is not symmetric any longer, therefore, we define a symmetric version

(2) $$\begin{array}{c} \tilde{p}(x,y)=p(x,y)\sqrt{\frac{v(x)}{v(y)}}\\ =\frac{k(x,y)}{\sqrt{v(x)v(y)}}\text{.} \end{array}$$

The corresponding matrix $\tilde{P}=\{\tilde{p}(x,y)\}$ is symmetric. Using the random walk formulation, the transition probability from *x *to *y *in *m *time steps is given by the *m*-th power of the matrix $\tilde{P}$. The element ${\tilde{p}}^{\left(m\right)}(x,\cdot )$ of the matrix ${\tilde{P}}^{m}$ can be thought of as a "bump" centered at *x *and of width proportional to *m*.

Then, the diffusion distance between two points *x *and *y *is defined as

(3) $$d_{m}^{2}(x,y)=\sum_{z\in X}|{\tilde{p}}^{\left(m\right)}(x,z)-{\tilde{p}}^{\left(m\right)}(y,z){|}^{2},$$

where $d_{m}^{2}(x,y)$ can be thought of as a distance between two bumps. Using eigen decomposition of $\tilde{P}$, we can expand $\tilde{p}(x,y)$ as

(4) $$\tilde{p}(x,y):=\sum_{i=1}^{n}\lambda_{i}^{2}\phi_{i}(x)\phi_{i}(y),$$

where {*λ_i_*} is the sequence of eigenvalues of $\tilde{P}$ (with *λ*_1 _= 1≥ |*λ_2_*|≥ ... |*λ*_*n*_|) and *ϕ_i _*are the corresponding eigenvectors. Therefore, for the elements of the matrix ${\tilde{P}}^{m}$ we obtain

(5) $${\tilde{p}}^{\left(m\right)}(x,y)=\sum_{i=1}^{n}\lambda_{i}^{2m}\phi_{i}(x)\phi_{i}(y),$$

Lastly, the eigenmap

(6) $$\Phi_{m}\left(x\right)=\left(\begin{array}{c} \lambda_{1}^{m}\phi_{1}(x)\\ \lambda_{2}^{m}\phi_{2}(x)\\ \vdots \end{array}\right),$$

defined by the eigenvalues and eigenvectors of $\tilde{P}$, is *diffusion map *in *m *time steps. The link between diffusion maps and distances can be summarized by the spectral identity

(7) $$\|\Phi_{m}(x)-\Phi_{m}{\left(y\right)\|}^{2}=d_{m}^{2}(x,y),$$

which means that the diffusion map Φ*_m _*embeds the data into a Euclidean space in which the Euclidean distance is equal to the diffusion distance *d_m _*in Eq. (3).

#### Diffusion kernels

The diffusion framework is essentially based on a Markov random walk on graphs. The first step views all data points on the shape as being the nodes of a graph *G*, in which two nodes *x *and *y *are connected by an edge. Once the graph is built, the weight of the edge in *G *is measured by the diffusion kernel *k*(*x*, *y*). In essence, the diffusion distance represents an average length of all the paths connecting node *x *and node *y *through *G *in a measure of *k*(*x*, *y*). Note that *k*(*x*, *y*) describes the relationship between *x *and *y*, and its choice should be guided by the application. Typically, the following Gaussian kernel is used

(8) $$k(x,y)=\exp(-d_{E}^{2}(x,y)/\sigma^{2}),$$

where *d_E_*(*x*, *y*) = ||*x *- *y*|| denotes the length of Euclidean distance between *x *and *y*, and *σ *indicates the variance of the Gaussian.

**Figure 3:**
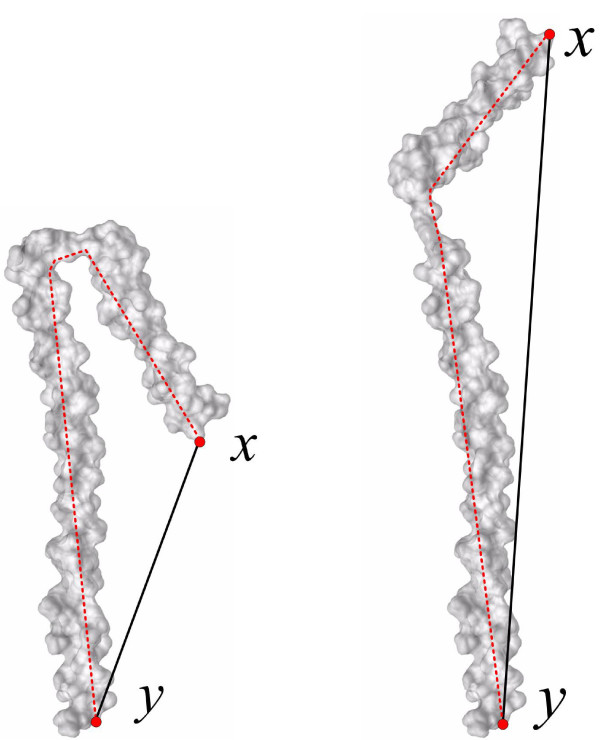
**Illustration of the inner distance**. The red dashed line denotes the inner distance, which is the shortest path within the shape boundary surface that connect two landmark points *x *and *y*. The right molecule is one deformation to the left one, and the relative change of the inner distances between the corresponding pair of points (e.g. *x *and *y*) during shape deformation are small. In contrast, the black bold line denotes the Euclidean distance defined as the length of the line segment between two landmark points *x *and *y*. Note that the Euclidean distance does not have the property of deformation invariant in contrast to the inner distance. This is because, the Euclidean distance does not consider whether the line segment crosses shape boundaries.

It is intuitive that if two nodes *x *and *y *are closer (more similar) in a sense of Euclidean distances, they are more likely transmitted to each other. However, as discussed in [13], the Euclidean distance as well as geodesic distance can not capture well the flexible molecular structures. It is easy to see that the Euclidean distance is sensitive to shape deformation. For instance, in Figure 3, the Euclidean distance, that is defined as the length of the line segment between two landmark points *x *and *y *(the black bold line), does not consider whether the line segment crosses shape boundaries.

### Inner distances

To overcome the disadvantage of the Euclidean distance used in the diffusion kernel *k*(*x*, *y*), we utilize the inner distance metric, as introduced in our previous studies [13]. Let *O *be a 3D shape as a connected and closed subset of ℝ^3^. We denote the boundary surface of *O *by *∂O*. Given two points *x*, *y *∈ *∂O*, the inner distance between *x *and *y *is defined as the length of the shortest path connecting *x *and *y *within *O*. For example, in Figure 3, the red dashed line denotes the inner distance, which is the shortest path within the shape boundary surface that connect two landmark points *x *and *y*. The right molecule is one deformation to the left one, and the relative change of the inner distances between the corresponding pair of points (e.g. *x *and *y*) during shape deformation are small. Note that the Euclidean distance does not have the property of deformation invariance in contrast to the inner distance.

Based on the above observation, we use the inner distance instead of the Euclidean distance *d_E_*(*x*, *y*) in Eq. (8) as follows

(9) $$k(x,y)=\exp(-d_{I}^{2}(x,y)/\sigma^{2}),$$

where *d_I_*(*x*, *y*) measures the length of inner distance between *x *and *y*. In other words, $\tilde{p}(x,y)$ based on Eq. (9) defines the transition probability from *x *and *y *in a sense of inner distances.

**Figure 4:**
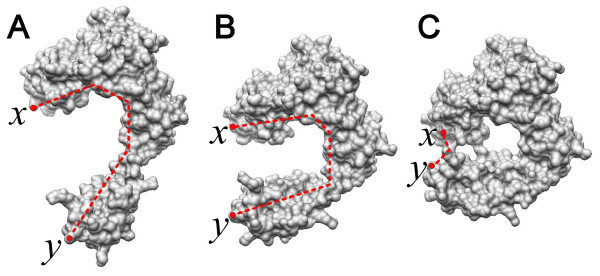
**Illustrating variation of inner distances for shape deformation with topological changes**. The red dashed lines denote the shortest paths within the shape boundary surface that connect two landmark points *x *and *y*. The object B is the deformation to the one A, and the relative changes of the inner distances between the corresponding pair of points (e.g. *x *and *y*) during the shape deformation are small. In contrast, although the object C is also a shape deformation of the object A, the topological change leads to a significant change for inner distance.

Figure 4 illustrates the variation of inner distance for shape deformation with topological changes, where the red dashed line segments denote the inner distance paths between two landmark points *x *and *y*. Note that the object B is a shape deformation of the object A. Intuitively, this example shows that the inner distance is insensitive to deformation, while the Euclidean distance does not have this property. The main advantage of inner distance is that it reflects shape structure and deformation without explicitly decomposing the shape into parts. Note that, although the object C in this figure is also a shape deformation of the object A, the topological change leads to a significant change of inner distance. The new diffusion distance could resolve the topology sensitivity problem for molecular shape deformation with the help of inner distances.

### Representations of molecular shapes

Molecular representations have various capabilities in terms of applications [36]. The surface-based representation of molecular shape is faithful to the actual physics of molecules and convenient for our application. A molecule can be defined by a set of spherical atoms whose exposed surface represents a molecular surface that defines the boundary of the molecular volume. In this paper, we consider the input molecule as a volumetric representation that is popularly used in biological research fields [9,37]. The volumetric model is composed of a uniform 3D lattice and it can be built in three steps as follows [12,38]. First we compute the Connolly surface (triangle mesh) of the molecule using the MSROLL program [39] with default parameters. Second, we place the triangle mesh in a 3D cubic grid of (such as 64 × 64 × 64) compactly. Third, each lattice point is assigned either 1 or 0; 1 for point inside the surface and 0 for outside. The inside point is denoted as object point and the outside point is denoted as background point. For each lattice point, there is a set of 26 neighbor points. An object point lies on the boundary if at least one of its 26 neighbors is a background point.

### Algorithm of DDSD

The DDSD algorithm for computing the diffusion distance shape descriptor of a volumetric object *O *is given as follows.

1. Sample uniformly *n *points *S *= {*q*_1_, *q*_2_, ..., *q_n_*} on the boundary surface *∂O *of *O *using Lloyd's algorithm of k-means clustering.

2. Calculate inner distances of all sample point pairs in *S*.

3. Calculate diffusion distances of all sample point pairs in *S*.

3.1. First, we define a weighted graph *G *over all sample points by connecting points *q_i_*, *q_j _*∈ *S*. Set edge weights equal to *k*(*q_i_*, *q_j_*) using Eq. (9).

3.2. Construct the transition probability matrix $\tilde{P}$ of the Markov chain by Eq. (1) and Eq. (2).

3.3. Compute the diffusion map Φ*_m _*at diffusion time *m *using Eq. (6).

3.4. Compute the Euclidean distance in Φ*_m _*equal to the diffusion distance *d_m_*.

4. Build the descriptor of the shape *O *as the histogram of values of diffusion distances using 128 bins.

The implementation details of algorithm will be given next.

#### Sampling the boundary surface

The volumetric shape of a molecule can be considered as a point array. The full boundary point set is too large to compute shape descriptor effectively. In order to save storage and computation costs, we choose a subset of of boundary points but preserve the characteristics of the shape in the same way to [13]. We compared two sample methods: random sampling and uniform sampling methods. With random sampling, every point in the boundary point set has an equal chance of inclusion in the sample set. The points picked out by the random sampling method can not yield an informative sample set. In this paper, we utilize the Lloyd's algorithm of k-means clustering for obtaining uniform sampling points on a molecular surface.

#### Calculating inner distances

In the second step, we compute inner distances of all sample point pairs in *S *using our recent work [38]. Here the inner distance is approximated by finding the shortest path distance in the graph, which is resolved using Dijkstra's algorithm. Dijkstra's algorithm is a graph search algorithm that solves the single source shortest path problem for a graph. In order to implement Dijkstra's algorithm more efficiently, Fibonacci heap is used as a priority queue. The code package of Dijkstra's algorithm was implemented in [40].

#### Computing diffusion distances

In the third step, our algorithm includes two parameters: *σ *and *m*. In our implementation, *σ *is the average of inner distances between all pairs of points in the shape; the time constant *m *= 50 is used for diffusion time.

The diffusion distance is considered as an average length of paths connecting two points on the shape within *m *steps (i.e. times). In fact, the diffusion distance is computed by embedding a 3D shape into a Euclidean space (i.e. diffusion map) in which the Euclidean distance is equal to the diffusion distance *d_m_*. The parameter *m *intuitively specifies the amount of "diffusion time" during which paths are explored to discover connectivity between sample points. If *m *is chosen too large, then only the eigenvector(s) with the lowest eigenvalues are considered during diffusion map, and the result is a distance measure with nice global geometry properties, but poor local geometry properties. On the other hand, if *m *is too small, then the diffusion process runs for only a short time, and the resulting distance is useful locally, but exhibits unexpected global geometry behavior [41]. For our application, the time constant *m *is suggested to be 50 for comparing molecular shapes. This selection (i.e. *m *= 50) has been used in [34,35] for non-rigid shape matching, which results in good results.

#### Building descriptors

A challenging aspect of measuring the similarity between two 3D shapes is to find a suitable shape descriptor that can be constructed and compared quickly, while still discriminating between similar and dissimilar shapes. Shape distribution [20] is the simplest and most widely used shape descriptor, which represents the shape descriptor of a 3D model as a probability distribution sampled from a shape function measuring geometric properties of the 3D model. In particular, the *distance histogram*, also called D2, is one example shape distribution, which represents the distribution of Euclidean distances between pairs of randomly selected points on the surface of a 3D model. The key idea of shape distribution is to transform an arbitrary 3D model into a parameterized function that can easily be compared with others. In our case, the domain of the shape function provides the parameterization (e.g., the D2 shape distribution is a function parameterized by distance), and random sampling provides the transformation. The primary advantage of shape distribution is its simplicity, where the shape matching problem is reduced to sampling, normalization, and comparison of probability distributions. In spite of its simplicity, the shape distribution is expected to be useful for discriminating the whole objects with different gross shapes, including invariance, robustness, efficiency, and generality. In general, the D2 histogram is computed by employing stochastic methods and it is formed by three steps: (1) first sampling uniformly random points from the shape surface, then (2) computing the Euclidean distance between the sampled point pairs, and finally (3) constructing a distance histogram by counting how many samples fall into each of fixed sized bins. From the histogram, we reconstruct a piecewise linear function which forms our representation for the shape distribution. In this paper, the main difference between our method and the original D2 is that we use diffusion distance in a sense of diffusion distance instead of Euclidean distance in [20].

After finishing computation of diffusion distances, we now convert a set of diffusion distances defined on the boundary of the object to a shape descriptor. This is done in a similar manner as D2 of shape distribution in [20]. Given *n *sample points, the number of diffusion distances of the shape is at most *n*^2^/2. We evaluate specifically *n*^2^/2 diffusion distance values from the shape distribution and construct a histogram by counting how many values fall into each of *n_bin _*fixed sized bins. In our experiments, we have found that using *n *= 500 samples and *n_bin _*= 128 bins yields shape descriptors with low enough variance and high enough resolution. Note that our distance histogram includes the step of normalization by aligning the maximum and minimal diffusion distance values, which yields invariance under rigid motions (e.g. scaling).

#### Measuring similarity

A shape descriptor can be used as an index in a database of shapes and it allows for very fast retrieval. Therefore, we have to choose an appropriate similarity measurement between two shape histograms to achieve accurate results. Osada et al. [20] investigated many standard ways of comparing two histograms, which include *L_p _*(*p *= 1, 2, ..., *∞*) norms, Bhattacharyya distance, and the *χ*^2 ^measurement. In our work, we have found that using different metrics on different descriptors may affect lightly the query results. Although in our experiments we tested all different types of metrics for each descriptor when possible, the metrics *L*_1 _and *L*_2 _norms are simple and usually give better results, where *L*_1 _norm is known as the Manhattan distance and *L*_2 _norm is the familiar Euclidean distance.

## Results

The DDSD algorithm presented in this paper has been implemented and incorporated into a shape search system of flexible molecules. The assistant program for computing inner distances is available from https://engineering.purdue.edu/PRECISE/IDSS. To demonstrate the abilities of DDSD, we test it on a benchmark containing abundant conformational changes of molecules.

### Deformation invariance of shapes

**Figure 5:**
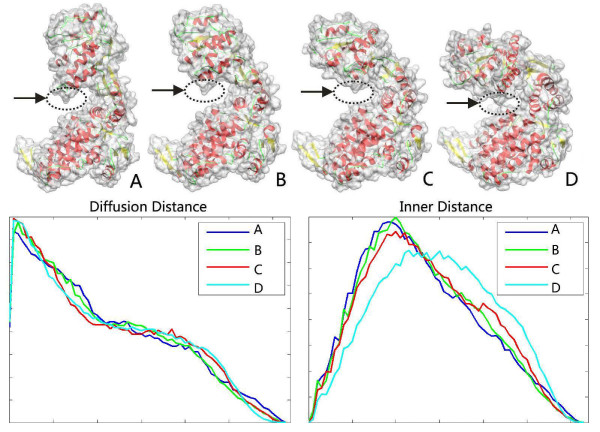
**The DD descriptor is compared to ID for the morph deformations between two conformations of GroEL: 1AON and 1KP8**. The first row shows the input four molecules with the same main chain orientation but with different surface shapes, where the arrows mark topological changes. The second row shows the DD and ID descriptors. Note that DD is not sensitive to shape deformation with topological changes, so four histograms are almost consistent; in contrast, ID is sensitive to topological changes.

**Figure 6:**
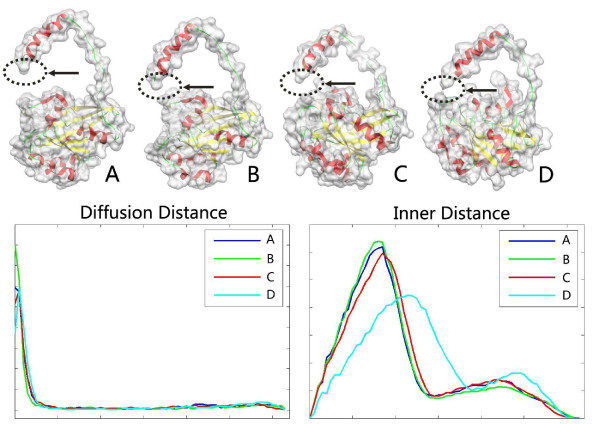
**Comparison between DD and ID**. The first row shows the four morph deformations between two conformations of Ran: 1BYU and 1RRP. The second row shows the DD and ID histogram. Note that DD is not sensitive to shape deformation with topological changes, so four histograms are almost consistent; however, ID is sensitive to topological changes.

In our first experiment, we select some representative molecules with conformational changes to show that the DD descriptor is insensitive to the shape deformation of flexible molecules. Figure 5 illustrates comparison among two methods: DD and ID. The first row shows the input four molecules (A, B, C and D) with the same main chain orientation but with different surface shapes. The four molecules are the morph deformation between two conformations of GroEL: 1AON and 1KP8. The regions in topological changes are highlighted. The second row shows the histogram comparison. Although the ID descriptors can keep almost consistent histogram for the first three deformed molecules, it is sensitive to shape topological changes for molecule D, which leads to the significant difference in histogram. In contrast, our DD descriptors remain largely consistent for the four deformed conformation shapes of the same protein. Figure 6 illustrates another comparison of DD and ID for the input four molecules with the same main chain orientation but with different surface shapes. The four molecules are the morph deformation between two conformations of Ran: 1BYU and 1RRP. The second row shows the histogram comparison. The ID descriptors is sensitive to topological changes of shapes, while our DD descriptors keep largely consistent for the four deformed conformation shapes of the same protein.

### MolMovDB benchmark

In order to evaluate the efficiency of the DDSD descriptor, we have incorporated our method into a simple search system for molecular shape comparison [13]. The presented method is tested on a benchmark of flexible molecules with conformations. This benchmark can be found in the Database of Macromolecular Movements (MolMovDB) [42]. MolMovDB contains a diverse set of molecules that display large conformational changes in proteins and other macromolecules (http://www.molmovdb.org/), also including the intermediate morphs. The original benchmark data set has the total 2,695 PDB files that are classified into 214 groups. The MolMovDB benchmark has been applied to molecular shape comparison, predicting protein structures, and so on [12,13,38,43]. The developed search system retrieves the tested database for molecules that most closely resemble the shape of a given query molecule in terms of their geometrical shapes. In the current search system, the user first chooses a query molecule and the program computes the similarity scores for all molecules in the tested database using the DDSD method described in this paper. The program then ranks all molecules in the database according to their similarity scores. The search system also contains our recent works [12,13], and the corresponding program and database are available. For the MolMovDB benchmark we have pre-computed all DD descriptors of queries on the database. The DD descriptors allow rapid search on the system because a molecular shape is compactly represented by a one-dimensional histogram. The query time to the data set of MolMovDB benchmark takes under a second if a query molecule is already transformed into the DD descriptor. For larger databases, more sophisticated indexing methods can further accelerate the performance. Even though this simple search system of flexible molecules is rather simple, it shows the potential of DDSD for flexible molecular shape comparison using a single computer and without the need of aligning the molecules before testing for similarity.

### Evaluation

To evaluate the effectiveness of the proposed descriptors, we compared the DD shape descriptor with some other shape descriptors in terms of the performance on retrieving similar molecular structures. In addition to three distance descriptors: ID, GD and ED, we also compare our descriptor to several known descriptors, which have been applied to search rigid shapes in many areas, such as engineering domain, computer graphics, and molecular shape comparison. They are as follows.

Spherical Harmonic Descriptor (SHD) [8,44] is a rotation invariant shape descriptor based on spherical harmonics.

Solid Angle Histogram (SAH) [45,46] measures the concavity and the convexity of a molecular surface. Histograms are computed based on a complete partitioning of the 3D space into disjoint cells which correspond to the bins of the histograms.

Shape Distribution (SD) [20] represents the shape descriptor as a probability distribution sampled from a shape function measuring the geometric properties of a 3D model. Here, we use the *D*2 shape measure for the triangle surface of a molecule.

#### Precision-recall curve

The standard evaluation procedures from information retrieval, namely *precision-recall *curves [9,47], are often used for evaluating the various shape distance descriptors, but other evaluation criteria also exist [24]. Precision-recall (PR) curves describe the relationship between precision and recall for an information retrieval method. Precision is a measure of exactness or fidelity, and it is the ratio of the relevant models retrieved to the retrieval size. Recall is a measure of completeness, and it is the fraction of the relevant models retrieved for a given retrieval size. Precision and recall are defined as follows:

(10) $$precision=\frac{TP}{TP+FP},$$

(11) $$recall\;=\frac{TP}{TP+FN},$$

where *TP*, the number of true positives, is the number of molecules that are included in the group that are the same as the query molecule correctly retrieved in the search; *FN*, the number of false negatives, is the number of molecules that included in the group same as the query molecule but missed in the search; *FP*, the number of false positives, is the number of molecules that are included in a different group from the query molecule but inaccurately retrieved in the search. Thus, the denominator in Eq. (10) is the total number of all members in a group and the denominator in Eq. (11) is the retrieval size. A perfect retrieval retrieves all relevant models consistently at each recall level, producing a horizontal line at precision = 1.0. However, in practice, precision decreases with increasing recall. The closer a precision-recall curve tends to the horizontal line at precision = 1.0, the better the information retrieval method. For more details, the reader can refer to [9,12,47].

**Figure 7:**
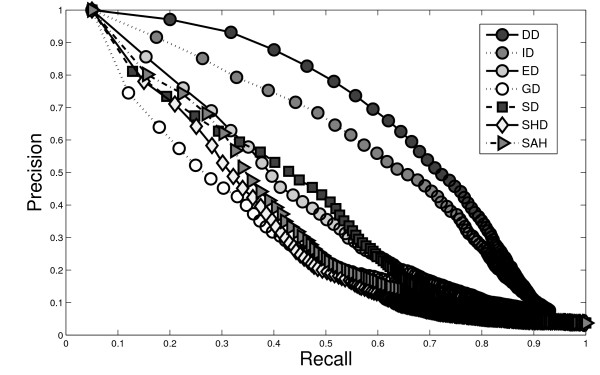
**Precision-recall curves compared with some existing shape descriptors for the MolMovDB database**. The results show that the DD method performs better than other shape descriptors.

Figure 7 shows the precision-recall curves for a subset of of the MolMovDB database by extracting the groups with topological changes. For precision-recall plots, the precision for each molecule or group is averaged using linear interpolation over the recall range. The results show that the DD method performs better than other descriptors for the MolMovDB database with vastly different conformation variations and topological changes.

#### Other measures

In addition to the precision-recall curves, we also evaluate other quantitative statistics for evaluation of retrieved results for the MolMovDB benchmark. Specifically, we compute E-measure and F-measure. E-measure is a composite measure of the precision and recall for a fixed number of retrieved results [25,48]. The intuition is that a user of a search engine is more interested in the first page of query results than in later pages, so this measure only considers the first 64 retrieved models for every query and calculates the precision and recall over those results. The E-measure [48] is defined as

$$E=\frac{2}{\frac{1}{precision}+\frac{1}{recall}},$$

where the maximum score is 1.0 and higher values indicate better results.

F-measure (also F-score) is a measure of a test's accuracy, and it is the harmonic mean of precision and recall. The F-measure is defined as

$$F=2\cdot \frac{precision\cdot recall}{precision+recall}.$$

This is also known as the *F*_1 _measure, because recall and precision are evenly weighted. We summarize the retrieval statistics in Table 1 for each method. Examining the results, we can see that the DD descriptor is higher in performance compared to other descriptors.

**Table 1:** Various quantitative measures evaluated on the MolMovDB database for different methods.

Methods	F-measure	E-measure
DD	41.87%	37.04%
ID	39.90%	35.83%
ED	31.04%	28.81%
GD	27.75%	26.42%
SD	31.11%	28.40%
SHD	26.06%	23.93%
SAH	27.86%	25.69%

The results show that our descriptor is higher in performance compared to others.

## Discussion

### Sampling rate

**Figure 8:**
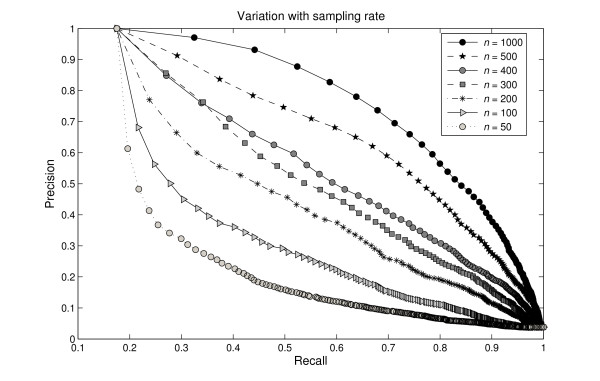
**Variation with increasing sampling rates for the MolMovDB database**. The precision-recall curves vary with *n *uniform sample points on the molecular surface for testing our method, where *n *∈ {50, 100, 200, 300, 400, 500, 1000}. The results suggest that the high sampling rate performs better than the low one.

In the first step of the DDSD algorithm, we sample uniformly *n *points on the molecular surface for computing diffusion distances. Figure 8 demonstrates the variation of precision-recall curves with increasing sampling rates *n *for the MolMovDB benchmark, where *n *∈ {50, 100, 200, 300, 400, 500, 1000}. The results suggest that the high sampling rate performs better than the low one. In this paper, we choose *n *= 500 sample points which yield shape descriptors with low enough variance and high enough resolution to be useful for our experiments. The higher sampling rate (e.g. *n *= 1000) could be used for pursuing the more accuracy. In addition, some other non-uniform sampling techniques such as curvature-adaptive clustering can also be applied to our work by restricting different stopping criteria, while this leads to an increase in computation time.

### Comparison with other work

#### Commute time

The diffusion distance metric introduced in this paper is similar to the average *commute time *by proposed in recent studies [32], in which Chennubhotla and Bahar presented the Markovian stochastic model of information diffusion developed for exploring the inter-residue communication in proteins. The process in [32] is controlled by transition probabilities for the passage/flow of information across the nodes, which in turn is based on the internode affinities derived from atom-atom contacts in the folded structures. Its key idea is to measure two basic quantities: *hitting time *and *commute time*, during Markov process of information transfer across the network of residues. Hitting time *H*(*j*, *i*) is the expected number of steps it takes to send information from residue *υ_i _*to residue *υ_j_*, and this may not be the same as *H*(*i*, *j*). Commute time *C*(*i*, *j*) is by definition the sum: *H*(*i*, *j*) + *H*(*j*, *i*). Hitting time has directionality, while commute time does not.

The diffusion distance is based on the eigenvalue decomposition of the Markov transition matrix, whereas the hit/commute times are derived from the graph Laplacian. Although both our work and [32] are essentially based on a Markov random walk on graphs, there are still some differences between the two works. They are as follows.

1. The application is different. The diffusion distance discussed in our paper deals with the surface-based representation of molecular shape, which is faithful to the actual physics of molecules and convenient for our application. We take advantage of diffusion distances for description of flexible molecular shape. Our goal is to discern the similarity between molecules for flexible molecular shape comparison, which is a challenging problem in query-retrieval in molecular databases. In contrast, the commute times in [32] deal with elastic network models for describing protein dynamics. It provides a tool for insights on the topological basis of communication in proteins and design principles for efficient signal transduction.

2. The definition is different. The diffusion distance in our work is defined by an average length of paths connecting two landmark points on the molecular surface in a sense of inner distances. In contrast, the commute times in [32], also named *resistance distance*, provide a metric with a means of estimating effective communication distances between residues *υ_i _*and *υ_j _*for the mean-square distance travelled by a random walk.

3. The methods are different. Our work is most related to Coifman's work [29], and the diffusion distance is based on the eigenvalue decomposition of the Markov transition matrix. However the commute times are derived from the graph Laplacian [32].

The central idea of our work is a combination of diffusion distance with inner distance. As we argued before in [13], the inner distance as a shape descriptor, is more appropriate for flexible molecular shape comparison. And by combing the inner distance and diffusion distance, we can define a new diffusion distance metric which can be considered as an average length of paths connecting two landmark points on the 3D shape in a sense of inner distances (inside the molecular shape), this can help resolving the topology sensitivity problem for molecular shape deformation.

#### 3D Zernike moments

More recently, Grandison et al. [22] proposed the use of 3D Zernike moments to compare molecular shapes. In contrast to the previous work [9,17], which used 3D Zernike moments for comparing the shapes of ligands and proteins, the enhanced method in [22] shows that not only molecular shapes but also that functions in 3D space can be represented and compared using this 3D Zernike technique. In particular, they explored the use of atomic displacement parameters and the diffraction precision index to build a 3D flexibility map of proteins and presented a novel approach to capture this information using 3D Zernike moments.

One appealing advantage in [22] is to capture the varying degrees of flexibility within molecules, especially protein structures. Their approach works well for small conformational changes in proteins, but performs poorly for large domain movements during protein deformation. The reason is that the flexibility map of proteins with small movements can be captured using 3D Zernike moments, but large motion results in missing density map [22].

In contrast, our method based on inner distance and diffusion distance can deal with the comparison between molecules even with large conformational changes. One of the limitations of our implementation is that it only utilizes geometry information of molecular shapes. Nonetheless other chemistry features, including atomic displacement parameters and the diffraction precision index used in Grandison et al.'s work [22], might be explored in the future. We will discuss the potential application later.

#### Laplace-Beltrami operator

In [26], Rustamov introduced a deformation invariant representation of surfaces, namely the GPS embedding, using the eigenvalues and eigenfunctions of the Laplace-Beltrami differential operator. The GPS representation embeds a surface into a high dimensional space, compared to geodesic multidimensional scaling (MDS) embedding (i.e. canonical forms) in [23], without using explicit geodesic distances. The GPS embedding provides a tool for processing of nonrigid shapes matching, in particular to local topology changes that is the same goal to our purpose. To demonstrate the application of shape matching, Rustamov first computed the GPS embedding of a given surface, and then found the *D*2 shape distribution in the embedding surface. Note that *D*2 shape distribution is the histogram of pairwise distances between the points uniformly sampled from the surface. In the way similar to Rustamov's method, our method also embeds a shape into a high dimensional space based on diffusion map, and find the *D*2 shape distribution in the embedding shape.

Although the core procedures (embedding + *D*2) between our method and Rustamov's method are similar, the embedding step in our method is different. In Rustamov's method, the GPS distance between two sample points can be considered as a particular diffusion distance in a sense of geodesic distances without explicit computation of geodesic distances. Bronstein et al. [49] have shown the relation between Rustamov's method and diffusion distances. It is worthwhile to emphasize our differences. The diffusion distance in our work is considered as an average length of paths connecting two landmark points on the molecular shape in a sense of inner distances.

Our argument for choosing inner distance (ID) instead of geodesic distance (GD) is that, like ID, GD is invariant to shape articulation deformation, but it is also invariant to a broader classes of deformation (bends) which preserves geodesic metric. The shape can be bent drastically and ends up with significant structural changes. Therefore, GD is weak in discriminating power for handling molecular shapes. On the other hand, ID is more appropriate since it reflects the essential invariant property for 3D shape articulation deformation. Meanwhile, ID is also sensitive to concavity/convexity changes of molecular shapes. This makes ID superior to GD as a flexible molecular shape representation. Although GD is insensitive to surface stretching or tearing, it remains invariant to all inelastic deformations as long as the deformation preserves geodesic (curve lengths) on the object boundary. From our experiments we also found that some molecules with one domain are often judged as similar to some ones with two or three domains when using GD descriptors. In the molecule database, GD can not give good search results as well as compared to ED.

#### Interior distance

It is worth noting that Rustamov et al. [27] more recently proposed the interior distance that is an interpolation of boundary distance in terms of two points inside the 3D shape. The interior distance is dependent on the choosing of metric for pairwise boundary distance and it propagates the boundary distance into inside using barycentric coordinate. The pairwise boundary distance can be geodesic distance, diffusion distance or even inner distance discussed in [13].

### Applications of molecular shape comparison

Molecular shape comparison (MSC) are playing an increasingly important role in a lot of biological activities, and DDSD presented in this paper can contribute to several future applications. One application is to search molecular databases for computer-aided drug design [2,3]. Its main goal is to identify compounds that are complementary to the site in molecular shape. An alternative technique for mining the information contained in these databases is MSC, which consists of searching the molecular database for compounds that most closely resemble the shape of a given query molecule. Some rigid MSC methods have been presented for this purpose [2,3,5]. For instance, Zauhar et al. [5] proposed a *shape signature *method for searching the Tripos fragment database and the NCI database. Ballester et al. [2,3] applied their fast MSC method to retrieve several compound databases, including the Vendor Database and an independent benchmark from DrugBank. However the molecular databases may include some information about the flexibility of the molecule with its possible conformations, the traditional rigid methods can not capture the shape similarity of flexible molecules well. The presented DDSD method may directly replace the existing rigid methods for search the molecular databases.

Another application is protein structure retrieval. There have been many protein structural comparison methods presented by computing the similarity scores, but most of them are based on protein structure alignment, such as DALI and CE. We recently presented a structural comparison method of flexible proteins using a robust statistics technique [38]. In contrast, the work in this paper can be applied to a search for similar protein structures. The main advantage is that the shape-based protein searching method does not produce any alignment between two proteins (i.e. correspondence between amino acids). The third application may be in cryo-electron microscopy (cryo-EM), where sheer shape comparison is important for example in discovery of high resolution structural homologues from cryo-EM maps [50,51]. The presented MSC method can overcome the different resolutions by considering both the geometrical shape and flexibility. For more explanation, the reader can refer to [13,51].

### Limitations

One of the limitations of our method is that it only utilizes geometry information of molecular shapes. Nonetheless chemistry features are also useful for matching as in protein-protein or protein-ligand (drug) docking/design. The option of including chemistry will reduce the number of false positive solutions and lead to better ranking. One possible solution is to combine other characteristics of a molecular surface into the DDSD computation. For instance, we can incorporate electrostatic potentials into the diffusion distance descriptor by considering a high dimensional sample point coordinate. In particular, we can describe a molecular boundary surface by a set of four-dimensional (4D) points {*q_i _*= (*x_i_*, *y_i_*, *z_i_*, *c_i_*)}, where *x_i_*, *y_i_*, and *z_i _*denote three geometry coordinates and *c_i _*is the corresponding charge value. The inner distance can be computed as the length of the shortest path between two 4 D points, so the diffusion distance can be considered as an average length of paths connecting two 4 D points on the molecular shape in a sense of inner distances. We plan to consider more chemical features into our descriptor in the future.

## Conclusions

In this paper, we describe a novel method, named DDSD, for molecular shape comparison (MSC). It is based on diffusion distances that is an average length of paths connecting two landmark points on the molecular shape in a sense of inner distances. The new method does not require previous alignment of the molecules being compared. We show that the new method is robust to deformation of flexible molecules, in particular to topological changes. In contrast, most existing MSC methods are effective for only comparing rigid objects and they can not capture well shape deformation of flexible molecules. We have evaluated and demonstrated the effectiveness of our method within a molecular search engine application for a benchmark on MolMovDB. Our method achieves good performance and retrieval results for different classes of flexible molecules. Furthermore, several potential applications for DDSD were discussed by replacing the conventional rigid shape descriptors.

We finally summarize the central idea of our work in this paper. Essentially our work is an improvement of inner distance with the additional step of calculating the diffusion distance and utilizing it as a descriptor instead of the inner distance. The merit of this article comes from the fact that this descriptor is an improvement over the inner distance, especially in the cases where structural topological changes.

The work in this paper only considers the problem of flexible molecular shape comparison using diffusion distance shape descriptor. However it is of interest to address the bigger question of to what degree molecular flexibility is involved in protein structural-functional relationships based on the retrieval results, and we leave this application to for our future work.

## Authors' contributions

YL generated the original idea, executed the research, and wrote the manuscript. QL implemented the idea, and GZ participated in the research. KR identified the underlying opportunity and basis for connecting diffusion to shape analysis. WB helped YL with the understanding of diffusion maps. All authors read and approved the final manuscript.
